# Development and validation of a novel nutrition-inflammation prognostic score for predicting 30-day mortality in critically ill stroke patients

**DOI:** 10.3389/fnut.2025.1658896

**Published:** 2025-09-03

**Authors:** Junzhuo Li, Jiajia Yang

**Affiliations:** ^1^Chongqing Jiulongpo District Yuzhou Road Street Community Health Service Center, Chongqing, China; ^2^Department of Rheumatology and Immunology, Center for Immune Ageing and Rejuvenation, The First Affiliated Hospital of Chongqing Medical University, Chongqing, China

**Keywords:** stroke, nutrition, prognosis, inflammation, biomarker

## Abstract

**Objectives:**

Malnutrition and systemic inflammation are common in critically ill stroke patients and contribute to poor outcomes. This study aimed to develop and validate a novel nutrition-inflammation prognostic score to predict critically ill stroke patients 30-day mortality and compare its performance with existing scores.

**Methods:**

In this retrospective study, a total of 926 critically ill stroke patients were included. The training cohort (*n* = 725) was used to develop the prognostic score. Feature selection was performed using three machine learning algorithms: LASSO, SVM-RFE, and Boruta. Four key biomarkers—high-sensitivity C-reactive protein, albumin, neutrophils, and D-Dimer—were identified. Based on these variables, a novel prognostic score, CAND, was constructed, visualized as a nomogram, and deployed as an online calculator. Cox regression analyses assessed the association between CAND defined high-risk groups and 30-day mortality, in comparison with existing nutrition-inflammation scores. The prognostic performance of CAND and these established scores was further evaluated using time-dependent receiver operating characteristic (ROC) curves, concordance index (C-index) and decision curve analysis (DCA). External validation was performed on 201 patients.

**Results:**

Higher CAND scores were independently associated with increased 30-day mortality risk in both the training cohort [hazard ratio (HR) = 3.273; 95% CI: 2.413–4.437; *P* < 0.001] and the validation cohort (HR = 3.608; 95% CI: 1.888–6.894, *P* < 0.001). CAND demonstrated strong discriminative ability and prognostic performance, with a C-index of 0.863 and time-dependent area under the curve(AUC) of 0.727 in the training cohort, and a C-index of 0.831 and AUC of 0.691 in the validation cohort. Compared to existing nutrition-inflammation scores, CAND consistently outperformed them in both cohorts, as further supported by time-dependent ROC and DCA.

**Conclusions:**

The CAND score, based on four objective biomarkers selected via machine learning, is a reliable and practical tool for early mortality risk stratification in critically ill stroke patients. Its application may inform timely clinical decision-making and targeted nutritional strategies.

## Introduction

The burden of stroke continues to rise with population aging (1). Despite advances in prevention and treatment, the risk of adverse clinical outcomes in patients with stroke still remains high, especially in critically ill patients (2). The clinical prognosis of stroke is influenced by multiple factors, among which systemic inflammation and nutritional status play pivotal roles. In critically ill stroke patients, inflammation and malnutrition commonly coexist (3). Systemic inflammation associated with stroke is considered a key driver of disease progression, and elevated inflammatory markers have been strongly linked to increased mortality risk (4–6). Likewise, nutritional status is also a significant prognostic determinant, with studies demonstrating that malnutrition is associated with poor clinical outcomes (7–9). Patients with impaired nutritional status generally exhibit worse prognoses.

Building upon the above evidence, developing a scoring system that comprehensively reflects both nutritional status and systemic inflammation may improve the accuracy of prognostic assessment in patients with stroke. In current clinical practice and research, various hematological parameters—such as albumin, hemoglobin, total cholesterol, lymphocytes, neutrophils, monocytes, and C-reactive protein—are widely used to evaluate nutritional and inflammatory status (10, 11). Based on these parameters, several nutrition-inflammation prognostic scores have been established, including the Prognostic Nutritional Index (PNI) (12), Controlling Nutritional Status (CONUT) (13), hemoglobin, albumin, lymphocyte, platelet (HALP) score, Naples Prognostic Score (NPS) (14), high-sensitivity modified Glasgow Prognostic Score (HS-m GPS) (15), and Prognostic Immune and Nutritional Index (PINI) (16). These scores are simple, objective, and easily calculated from routine blood tests, making them practical for use in critically ill patients. However, to date, no nutrition-inflammation prognostic score has been specifically developed for critically ill patients with stroke. While ICU scores like Sequential Organ Failure Assessment (SOFA) help assess illness severity, they are not tailored to stroke. Stroke patients may have preserved organ function early on, despite neurological damage and inflammation. Moreover, such tools overlook the critical role of malnutrition-inflammation interplay in stroke outcomes. A stroke-specific score integrating these factors is needed to improve risk stratification.

Therefore, this study was designed with the following objectives: (1) to utilize machine learning algorithms to identify nutrition- and inflammation-related hematological biomarkers that are closely associated with stroke prognosis; (2) to develop a novel composite nutrition-inflammation Prognostic score specifically tailored for patients with stroke based on the selected biomarkers; and (3) to compare the prognostic performance of the newly constructed score with that of existing nutrition-inflammation scoring systems, in order to validate its effectiveness in predicting mortality in critically ill patients with stroke.

## Methods

### Study description

This single-center, retrospective observational study was approved by the Ethics Committee of the First Affiliated Hospital of Chongqing Medical University (Approval No. 2023.049) and conducted in accordance with the Declaration of Helsinki. Due to its retrospective nature, informed consent was waived.

### Patient population

The admission criteria for critically ill stroke patients in the Neurological Intensive Care Unit (NCU) were defined as a Glasgow Coma Scale (GCS) score < 12 and/or an Acute Physiology and Chronic Health Evaluation II (APACHE II) score >15 (17). In addition, patients were admitted if they presented with major cerebral infarctions involving more than two-thirds of the middle cerebral artery territory; extensive cerebellar infarctions affecting the territories of the superior cerebellar artery, anterior inferior cerebellar artery, or posterior inferior cerebellar artery—with or without altered consciousness or significant mass effect on CT imaging; locked-in syndrome; top-of-the-basilar syndrome; critical brainstem infarctions; severe intracerebral hemorrhages (hematoma >20 ml supratentorial, >3 cm cerebellar, or >5 ml brainstem). For patients with multiple NCU admissions during the study period, only data from the first admission was included in the analysis.

From January 2018 to January 2023, stroke patients admitted to the Neurocritical Care Unit were retrospectively screened. Inclusion criteria: (1) age ≥18 years; (2) diagnosis of either hemorrhagic or ischemic stroke based on clinical presentation and neuroimaging findings, according to the International Classification of Diseases, 10th Revision (ICD-10, codes I60–I69). Patients were excluded if they (1) admission to the NCU for < 48 h or (2) had incomplete data. Admissions from January 2018 to December 2021 formed the training cohort; those from January 2022 to January 2023 comprised the validation cohort.

### Data collection

Data were retrospectively collected through a review of electronic medical records. Demographic characteristics encompassed age, sex, weight, height, living situation (living alone/living with family or carer or residential care), smoking status, and alcohol consumption (ever/never). Clinical data included stroke subtype (ischemic or hemorrhagic), comorbidities [hypertension, diabetes mellitus, chronic obstructive pulmonary disease (COPD), and coronary artery disease], and admission scores: the National Institutes of Health Stroke Scale (NIHSS) (18), GCS (19), and APACHE II score (20). Laboratory parameters were collected within 24 h of admission and included fibrinogen, D-Dimer, hematocrit, erythrocyte count, hemoglobin, platelet count, platelet distribution width (PDW), neutrophils, lymphocytes, monocytes, red cell distribution width (RDW), procalcitonin, albumin, urea, blood urea nitrogen (BUN), total cholesterol, triglycerides, high-density lipoprotein cholesterol (HDL-C), low-density lipoprotein cholesterol (LDL-C), high-sensitivity C-reactive protein (HS-CRP), and mean corpuscular volume (MCV).

### Calculation and classification of nutritional-inflammatory prognostic scores

PNI, CONUT, HALP, NPS, PINI, and HS-m GPS were calculated based on the collected laboratory data. The cut-off points of each score were determined based on the existing literature. Patients were classified as high risk based on the following thresholds: CONUT score ≥2 (21), PNI < 45 (22), HALP score < 56.8 (23), NPS score ≥1 (24), HS-m GPS ≥1 (25), and PINI < 3 (16) (Supplementary Table 1).

### Outcomes

The outcome was 30-day all-cause mortality after stroke. Patients were followed up via telephone interviews or clinical visits at 30 days poststroke. Patient outcomes were obtained from follow-up records.

### Inflammatory and nutritional biomarkers with specific predictive value for 30-day mortality were identified using machine learning

Three commonly used machine learning algorithms were employed to select nutritional-inflammatory biomarkers associated with mortality risk in training cohort. The least absolute shrinkage and selection operator (LASSO), which can be used for both continuous and categorical variables, was employed to enhance model prediction accuracy and interpretability. The optimal penalty parameter (lambda, λ) was determined via 10-fold cross-validation using the minimum mean cross-validated error criterion. The optimal lambda value selected was 0.022. Support Vector Machine-Recursive Feature Elimination (SVM-RFE), a technique based on the principles of support vector machines, was implemented to recursively eliminate features with the goal of identifying the most informative variables for classification. We used a radial basis function (RBF) kernel with a regularization parameter (C) set to 1. The optimal feature subset was identified by 10-fold cross-validation, minimizing classification error. The Boruta algorithm, a supervised feature selection method, was utilized to identify all relevant variables associated with the classification outcome (26, 27). The number of trees was set to 500, and the maximum number of runs was set to 100 to ensure algorithm stability and convergence.

To ensure that only the most robust and relevant features were included in the final model, we selected the intersection of variables identified by all three algorithms as the final feature set. This approach was adopted to improve model accuracy and generalizability while minimizing the risk of overfitting and inclusion of irrelevant predictors. Venn diagrams were used to visualize the overlapping biomarkers identified by the three machine learning methods.

To enhance model interpretability, SHapley Additive exPlanations (SHAP) values were calculated for each biomarker feature. SHAP values quantify the marginal contribution of individual features to the model's predictions, allowing identification of the most influential variables. To address potential collinearity, pairwise Pearson correlation matrices were constructed. Variables with correlation coefficients >0.8 were excluded to ensure each feature's independent contribution to the outcome (28).

### Construction of a novel nutritional-inflammatory prognostic score

A prognostic score was constructed based on selected nutritional-inflammatory biomarkers. A multivariable logistic regression model was constructed in the training cohort to develop a nomogram. The regression coefficients were used to assign variable weights. To enhance clinical accessibility, the nomogram was further implemented as an interactive online calculator to enable individualized risk estimation for critically ill stroke patients.

### Statistical analysis

Continuous variables were reported as mean ± SD or median (IQR), and compared using Student's *t*-test or Mann–Whitney *U*-test, as appropriate. Categorical variables were expressed as counts (percentages) and analyzed using χ^2^ or Fisher's exact tests. The novel prognostic score was dichotomized into high- and low-risk groups based on the optimal cut-off determined by maximally rank statistics. Survival differences by risk group—based on the novel and comparator scores (PNI, CONUT, HALP, NPS, HS-m GPS, PINI)—were assessed via Kaplan–Meier curves and log-rank tests. Univariate and multivariate Cox regression analyses evaluated associations with 30-day mortality. Three models were built: unadjusted (Model 1), adjusted for age and sex (Model 2), and further adjusted for demographic characteristics (living situation, smoking status, and alcohol consumption) and clinical factors (stroke subtype, comorbidities, NIHSS, GCS, and APACHE II score) (Model 3). The discriminative performance of the novel prognostic score and comparator scores in predicting 30-day mortality was assessed using time-dependent receiver operating characteristic (ROC) curves and concordance index (C-index). Decision curve analysis (DCA) assessed net clinical benefit of each scoring system. Integrated discrimination improvement (IDI) and net reclassification improvement (NRI) quantified improvement in predictive accuracy. External validation was performed in an independent cohort. To assess the adequacy of the validation cohort sample size, a *post hoc* power analysis was conducted based on the observed hazard ratio, event rate, and cohort size. Analyses used R software (v4.4.1); *P* < 0.05 was considered significant.

## Result

### Patient characteristics

A total of 926 patients were included, with 725 in the training cohort and 201 in the validation cohort. The patient recruitment process and novel prognostic score development are illustrated in Supplementary Figure 1, and the baseline characteristics are detailed in Table 1. Compared to survivors, those who succumbed to their conditions were older, more often female, and had higher rates of ischemic stroke, diabetes, COPD, higher NIHSS and APACHE II scores, and lower GCS (Table 1).

**Table 1:** Characteristics of the patients.

**Variables**	**Total**	**Training cohort**	**Validation cohort**	***P*–value**	**Mortality**	**Survival**	***P*–value**
*N*	926	725	201		241	685	
**Demographic characteristics**							
Age, years, median (IQR)	72 (60–80)	72 (61, 79)	74 (57, 84)	0.115	75 (67–83)	70 (58–78)	< 0.001
Female, *n* (%)	363 (39.2%)	285 (39.3%)	78 (38.8%)	0.897	114 (47.3%)	249 (36.4%)	0.003
Live alone, *n* (%)	193 (20.9%)	155 (21.4%)	38 (18.9%)	0.445	57 (23.7%)	136 (19.9%)	0.212
Smoking, *n* (%)	260 (28.1%)	204 (28.1%)	56 (27.9%)	0.938	67 (27.8%)	193 (28.2%)	0.911
Alcohol consumption, *n* (%)	318 (34.4%)	246 (33.9%)	72 (35.8%)	0.618	94 (39.0%)	224 (32.7%)	0.076
BMI, kg/m^2^, median (IQR)	23.50 (21.35–25.71)	23.44 (21.36, 25.40)	23.8 (21.30–25.93)	0.309	22.83 (20.55–25.14)	23.66 (21.57–25.71)	0.001
**Clinical information**							
Ischemic stroke	681 (73.6%)	533 (73.5%)	148 (73.6%)	0.974	51 (21.2%)	194 (28.3%)	0.030
Hypertension, *n* (%)	646 (69.8%)	515 (71.0%)	131 (65.2%)	0.109	172 (71.4%)	474 (69.2%)	0.528
Diabetes, *n* (%)	271 (29.3%)	234 (32.3%)	37 (18.4%)	0.000	99 (41.1%)	172 (25.1%)	< 0.001
Coronary heart disease, *n* (%)	195 (21.1%)	173 (23.9%)	22 (10.9%)	0.000	56 (23.2%)	139 (20.3%)	0.335
COPD, *n* (%)	85 (9.2%)	73 (10.1%)	12 (6.0%)	0.075	32 (13.3%)	53 (7.7%)	0.010
NIHSS, median (IQR)	16 (11–19)	16 (11, 19)	15 (10, 18)	0.438	19 (15–22)	15 (9–18)	< 0.001
GCS, median (IQR)	8 (7–11)	8 (7, 11)	9 (7, 11)	0.217	7 (6–8)	9 (8–12)	< 0.001
APACHE II, median (IQR)	16 (13–20)	16 (13, 20)	17 (14, 20)	0.883	19 (16–24)	16 (12–19)	< 0.001
**Measurements**							
Albumin, median (IQR)	39 (36–43)	39 (36, 43)	40 (37–43)	0.164	38 (35–42)	40 (37–44)	< 0.001
Hemoglobin	134.5 (120–146)	135 (120, 146)	134 (123–149)	0.544	133 (115–145)	135 (123–147)	0.012
Total cholesterol, mmol/L, median (IQR)	4.3 (3.66–5.18)	4.30 (3.67, 5.18)	4.28 (3.63–5.02)	0.458	4.39 (3.51–5.3)	4.3 (3.76–5.17)	0.560
Triglycerides, mmol/L, median (IQR)	1.21 (0.85–1.73)	1.21 (0.85, 1.7)	1.21 (0.85–1.81)	0.877	1.19 (0.92–1.81)	1.21 (0.83–1.68)	0.087
LDL–C, mmol/L, median (IQR)	2.69 (1.99–3.33)	2.71 (2.02, 3.43)	2.48 (1.86–3.18)	0.038	2.6 (1.83–3.19)	2.7 (2.03–3.39)	0.125
HDL–C, mmol/L, median (IQR)	1.24 (1.02–1.44)	1.24 (1.02, 1.43)	1.25 (1.03–1.44)	0.803	1.23 (0.99–1.44)	1.25 (1.04–1.43)	0.280
Lymphocyte, 10^9^/L, median (IQR)	1.11 (0.81–1.56)	1.09 (0.81, 1.55)	1.18 (0.78–1.68)	0.335	1.00 (0.77–1.34)	1.18 (0.825–1.61)	< 0.001
Neutrophils, 10^9^/L, median (IQR)	7.32 (5.09–10.17)	7.36 (5.06, 10.16)	7.23 (5.21–10.45)	0.996	9.53 (6.77–12.17)	6.77 (4.78–9.8)	< 0.001
Mononuclear, 10^9^/L, median (IQR)	0.56 (0.34–0.78)	0.56 (0.33, 0.81)	0.54 (0.40–0.72)	0.700	0.63 (0.41–0.84)	0.53 (0.34–0.75)	0.001
Platelet, 10^9^/L, median (IQR)	191 (154–230)	190 (153, 228)	197 (157–236)	0.165	186 (152–233)	192 (154–230)	0.415
HS–CRP, mg/L, median (IQR)	6.9 (2.06–18.78)	7.08 (2.12, 18.28)	6.86 (1.47–19.8)	0.576	13.8 (5.47–20)	4.66 (1.55–12.72)	< 0.001
Fibrinogen, g/L, median (IQR)	3.11 (2.49–3.95)	3.10 (2.49, 3.94)	3.21 (2.5–4.06)	0.389	3.27 (2.61–4.09)	3.09 (2.45–3.94)	0.131
Procalcitonin, ng/ml, median (IQR)	0.05 (0.03–0.12)	0.05 (0.03, 0.12)	0.05 (0.03–0.1)	0.174	0.06 (0.04–0.23)	0.05 (0.03–0.085)	< 0.001
D–Dimer, mg/L FEU, median (IQR)	1.9 (1.13–3.63)	1.96 (1.21, 3.43)	1.79 (0.91–3.95)	0.200	4.32 (2.16–6.64)	1.65 (0.99–2.5)	< 0.001
Hematocrit, %, median (IQR)	40.95 (37.08–44.23)	40.8 (37.05, 44.2)	41.4 (37.05–44.3)	0.796	40.9 (35.9–44.5)	41.1 (37.2–43.85)	0.719
Erythrocyte count, 10^12^/L, median (IQR)	4.47 (4.09–4.91)	4.47 (4.09, 4.89)	4.45 (4.17–5.03)	0.389	4.47 (4.04–4.86)	4.47 (4.1–4.95)	0.709
PDW, fl, median (IQR)	13.3 (11.7–15.33)	13.3 (11.7, 15.3)	13.4 (11.7–15.45)	0.598	13.3 (11.6–15)	13.3 (11.7–15.4)	0.715
RDW, %, median (IQR)	13.3 (12.7–14.1)	13.4 (12.7, 14.1)	13.1 (12.6–13.8)	0.005	13.4 (12.85–14.1)	13.3 (12.6–15.4)	0.064
BUN, mmol/L, median (IQR)	70 (58–87)	70 (58.5, 87)	70 (58–86)	0.798	73 (63–102)	69 (58–83)	< 0.001
Urea, mmol/L, median (IQR)	5.9 (4.6–7.8)	5.9 (4.6, 7.7)	5.9 (4.6–7.8)	0.883	6.2 (4.95–8.6)	5.6 (4.6–7.4)	< 0.001
McV, fl, median (IQR)	92.2 (89.6–95.71)	92.29 (89.69, 95.63)	91.9 (89.05–96.15)	0.489	91.8 (90–95.85)	92.32 (89.14–95.7)	0.421

NIHSS, National Institutes of Health Stroke Scale; GCS, Glasgow Coma Scale; APACHE II, acute physiology and chronic health evaluation scoring system II; COPD, chronic obstructive pulmonary disease; BMI, body mass index; HS-CRP, high sensitivity C-reactive protein; LDL-C, low-density lipoprotein cholesterol; HDL-C, high-density lipoprotein cholesterol; PDW, platelet distribution width; RDW, red cell distribution width; BUN, blood urea nitrogen; MCV, mean corpuscular volume.

### Feature selection

**Figure 1:**
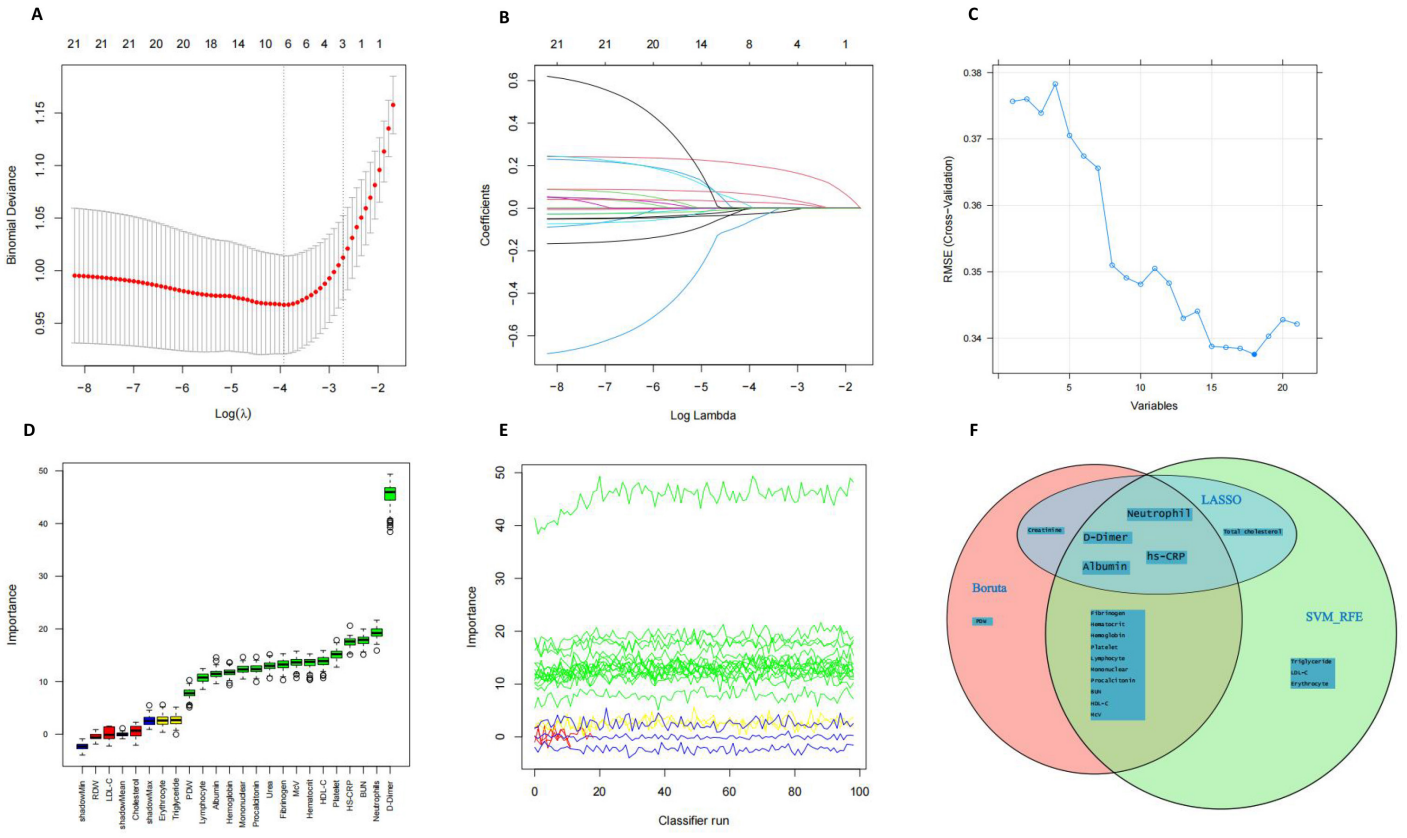
Feature Selection. **(A, B)** Feature selection performed using the Lasso regression algorithm. **(C)** Feature selection based on Support Vector Machine–Recursive Feature Elimination (SVM-RFE). **(D, E)** Feature selection using the Boruta algorithm. **(F)** Venn diagram illustrates the overlap of features selected by the three machine learning approaches.

All patients from the training dataset were utilized for feature selection and novel score development. A comprehensive screening of 21 potential prognosis-related nutritional and inflammatory hematological parameters was conducted using LASSO (Figures 1A, B), SVM-RFE (Figure 1C), and Boruta (Figures 1D, E) algorithms. Ultimately, four significant factors were consistently identified across all methodologies as predictors of 30-day mortality: HS-CRP), albumin, neutrophils, and D-Dimer (Figure 1F). SHAP analysis revealed that D-Dimer, HS-CRP, neutrophils, and albumin had the high impact on model prediction, each with a mean absolute SHAP value exceeding 0.318 (Supplementary Figure 2). These findings reinforce the clinical relevance of the selected biomarkers. Furthermore, as detailed in Supplementary Figure 3, none of the pairwise Pearson correlation coefficients among these features exceeded 0.8, indicating a lack of collinearity.

### CAND score development

**Figure 2:**
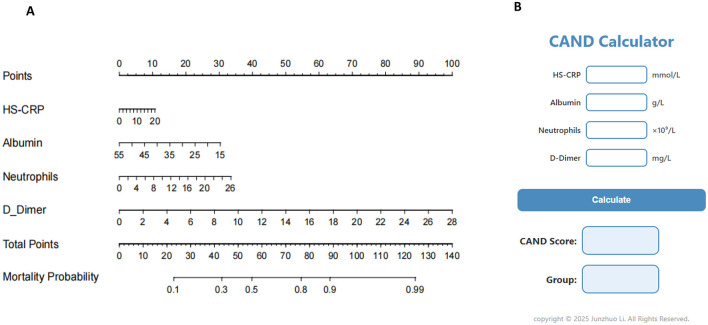
Nomogram and calculator for predicting mortality of critically ill patients. **(A)** Nomogram. **(B)** CAND calculator.

To provide healthcare professionals and researchers with a clear and practical prognostic tool, a nomogram was constructed based on four nutritional and inflammatory biomarkers—HS-CRP, albumin, neutrophils, and D-Dimer—identified through machine learning, to predict 30-day mortality. The results of the multivariable logistic regression analysis are presented in Supplementary Table 2. A visual representation of the nomogram is presented in Figure 2A. Each component of the index was assigned a weighted score based on its prognostic importance, as determined from the nomogram. Specifically, HS-CRP was allocated 10.7 points, albumin was allocated 30.3 points, neutrophils were allocated 33.5 points, and D-Dimer was allocated 100 points.

Based on these weights, we formulated the HS-CRP–Albumin–Neutrophils–D-Dimer nutrition-inflammation prognostic score (CAND) as follows: CAND = (10.7/20) ^*^ HS-CRP + (30.3/55–15) ^*^ (55-Albumin) + (33.5/26) ^*^ Neutrophils + (100/28) ^*^ D-Dimer, equating to CAND = 0.54^*^HS-CRP-0.76^*^Albumin + 1.29^*^Neutrophils + 3.57^*^D-Dimer + 41.7. To facilitate its clinical utility, we developed a web-based calculator (CAND Calculator) derived from the nomogram model, enabling users to input relevant laboratory values and instantly obtain the CAND score (Figure 2B). The calculator also provides risk stratification for malnutrition based on the CAND score. The optimal cut-off value for CAND was identified as 45 points (Supplementary Figure 4), with scores ≥45 indicating high risk and < 45 indicating low risk of adverse outcomes.

### Prevalence of high nutritional-inflammatory risk

According to assessments using CAND, PNI, CONUT, HALP, NPS, PINI, and HS-m GPS, 236 (25.5%), 401 (43.3%), 681 (73.5%), 792 (85.6%), 808 (87.3%), 156 (16.8%), and 627 (67.7%) patients, respectively, were identified as being at risk of malnutrition (Table 2). Except for the HALP score, patients classified as having high nutritional-inflammatory risk exhibited significantly higher mortality rates compared to those at low risk (*P* < 0.05).

**Table 2:** Nutritional-inflammatory risk and mortality across different scores.

**Positive nutrition risk screening**	**Total (*n* = 926)**	**Training cohort (*n* = 725)**	**Validation cohort (*n* = 201)**	***P*-value**	**Mortality (*n* = 241)**	**Survival (*n* = 685)**	***P*-value**
CAND	236 (25.5%)	184 (25.4%)	52 (25.9%)	0.888	141 (58.5%)	95 (13.9%)	< 0.001
PNI	401 (43.3%)	327 (45.1%)	74 (36.8%)	0.036	131 (54.4%)	270 (39.4%)	< 0.001
CONUT	681 (73.5%)	543 (74.9%)	138 (68.7%)	0.076	194 (80.5%)	487 (71.0%)	0.004
HALP	792 (85.6%)	623 (85.9%)	169 (84.1%)	0.509	214 (88.8%)	578 (84.4%)	0.094
NPS	808 (87.3%)	654 (90.2%)	154 (76.6%)	0.000	221 (91.7%)	587 (85.7%)	0.016
PINI	156 (16.8%)	124 (17.1%)	32 (15.9%)	0.692	57 (23.7%)	99 (14.5%)	0.001
HS-m GPS	627 (67.7%)	493 (68.0%)	134 (66.7%)	0.721	202 (83.8%)	425 (62.0%)	< 0.001

CONUT, controlling nutritional status score; PNI, prognostic nutritional index; HALP, albumin, lymphocyte, platelet score; NPS, naples prognostic score; HS-m GPS, high-sensitivity modified glasgow prognostic score; PINI, prognostic immune nutritional index.

### Comparison of the prognostic ability of nutrition-inflammation scores in both the training and validation cohorts

Kaplan–Meier survival analyses were conducted to evaluate the association between nutrition-inflammation risk, as stratified by various scores, and all-cause mortality (Supplementary Figures 5, 6). In both the training and validation cohorts, patients identified as high risk based on the CAND, PNI, PINI, and HS-m GPS scores demonstrated significantly poorer survival compared to their low-risk counterparts (*P* < 0.05).

**Figure 3:**
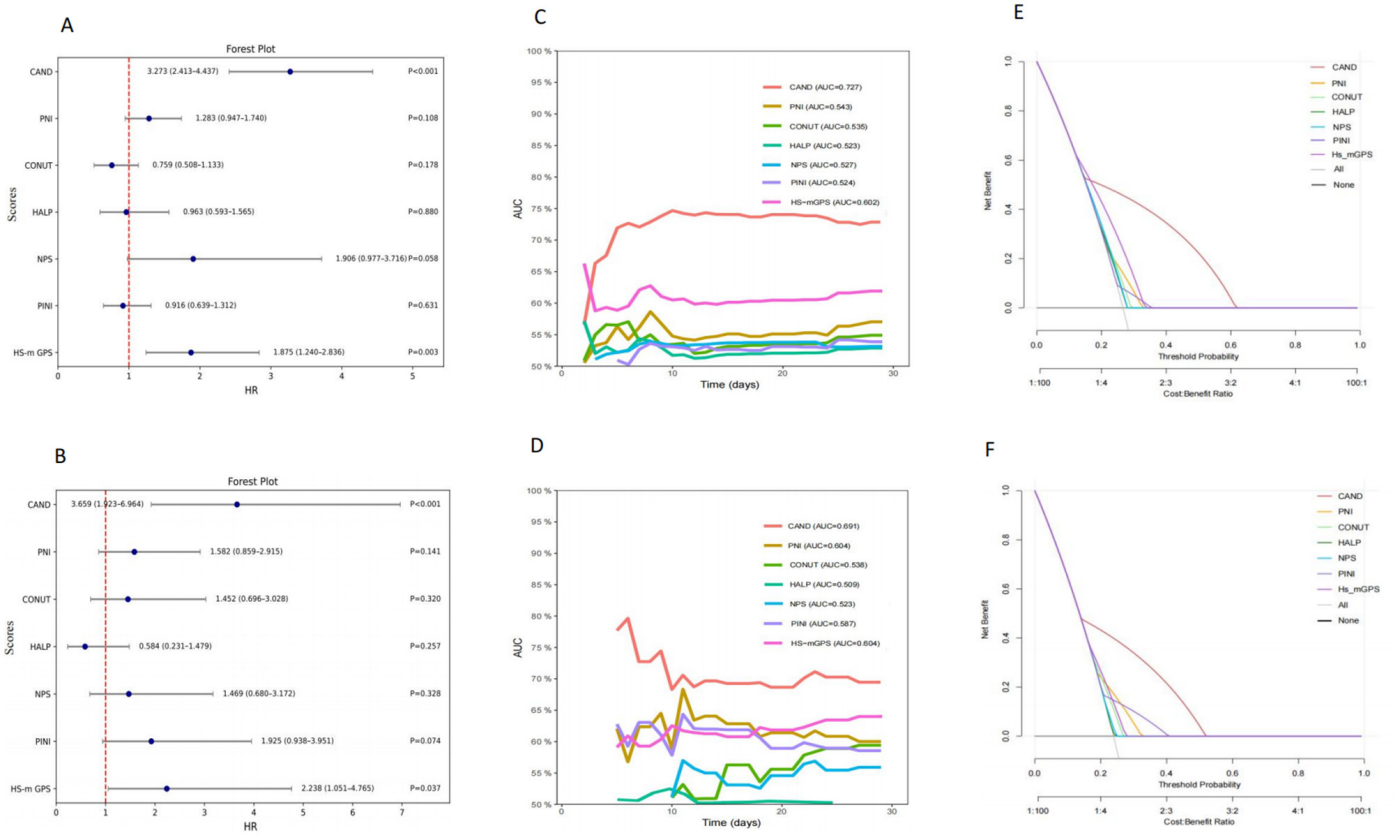
Comparative analysis of the prognostic performance of CAND and other nutritional-inflammation scores. **(A)** Forest plot of multivariate Cox regression analysis in the training cohort. **(B)** Forest plot of multivariate Cox regression analysis in the validation cohort. **(C)** Time-dependent ROC curves for predicting 30-day mortality in the training cohort. **(D)** Time-dependent ROC curves for predicting 30-day mortality in the validation cohort. **(E)** Decision curve analysis for different scores in the training cohort. **(F)** Decision curve analysis for different scores in the validation cohort.

Multivariate analysis identified CAND and HS-m GPS as independent predictors of 30-day mortality. In the training cohort, CAND showed an HR of 3.273 (95% CI: 2.413–4.437, *P* < 0.001), and 3.608 (95% CI: 1.888–6.894, *P* < 0.001) in the validation cohort. HS-m GPS showed HRs of 1.875 (95% CI: 1.240–2.836, *P* = 0.003) and 2.201 (95% CI: 1.035–4.683, *P* = 0.037), respectively (Supplementary Table 5; Figures 3A, B). Time-dependent ROC curves and C-index confirmed CAND's superior discriminative performance over PNI, CONUT, HALP, HS-m GPS, PINI, and NPS in both cohorts (Table 3, Figures 3C, D). DCA further showed CAND offered the highest net clinical benefit (Figures 3E, F). IDI and NRI analyses demonstrated that CAND improved risk reclassification by 25.2% and discrimination by 9.8% (both *P* < 0.001) in the training cohort, and by 19.4% (*P* = 0.0332) and 11.3% (*P* < 0.001) in the validation cohort (Supplementary Table 5). A *post hoc* power analysis of the validation cohort indicated a statistical power of 0.85, suggesting acceptable discriminatory ability.

**Table 3:** Discriminative performance of nutrition-inflammation prognostic score in the training validation cohorts.

**Positive risk screening**	**Training cohort (*****n*** = **725)**		**Validation cohort (*****n*** =**201 )**	
	**AUC, 95% CI**	**C-index, 95% CI**	**AUC, 95% CI**	**C-index, 95% CI**
CAND	0.727 (0.688–0.765)	0.863 (0.827–0.898)	0.691 (0.617–0.772)	0.831 (0.746–0.916)
PNI	0.543 (0.503–0.585)	0.615 (0.547–0.683)	0.604 (0.535–0.683)	0.7 (0.576–0.825)
CONUT	0.535 (0.502–0.567)	0.621 (0.533–0.71)	0.538 (0.454–0.613)	0.605 (0.44–0.771)
HALP	0.523 (0.499–0.549)	0.616 (0.503–0.729)	0.509 (0.432–0.561)	0.507 (0.314–0.701)
NPS	0.527 (0.506–0.548)	0.686 (0.548–0.825)	0.523 (0.44–0.582)	0.578 (0.402–0.754)
PINI	0.524 (0.491–0.560)	0.597 (0.516–0.678)	0.587 (0.519–0.671)	0.756 (0.629–0.883)
HS-m GPS	0.602 (0.572–0.633)	0.761 (0.687–0.834)	0.604 (0.526–0.667)	0.675 (0.523–0.828)

CONUT, controlling nutritional status score; PNI, prognostic nutritional index; HALP, albumin, lymphocyte, platelet score; NPS, Naples Prognostic Score; HS-m GPS, high-sensitivity modified Glasgow Prognostic Score; PINI, prognostic immune nutritional index.

## Discussion

In this study, we developed a novel prognostic score, CAND, based on nutrition-inflammation related blood biomarkers, including HS-CRP, albumin, neutrophils, and D-Dimer. We investigated the associations between CAND, along with other established nutrition-inflammation scores (PNI, CONUT, HALP, HS-m GPS, NPS, and PINI), and 30-day all-cause mortality in critically ill adults with stroke to evaluate their prognostic value. Kaplan–Meier survival analyses and multivariate Cox regression revealed that CAND and HS-m GPS were independent predictors of mortality in both the training and validation cohorts. Among these, CAND demonstrated superior predictive performance for mortality and validation cohort, outperforming other nutritional-inflammation scores. Furthermore, C-index, time-dependent ROC and DCA curve analysis confirmed that CAND had a higher prognostic accuracy compared to the other indicators. These findings suggest that CAND may serve as a valuable tool for assessing nutritional-inflammatory status and predicting outcomes in patients with stroke-related critical illness.

CAND consists of four components—HS-CRP, albumin, neutrophils, and D-dimer, which together reflect both nutritional and inflammatory status. HS-CRP is a refined assay of C-reactive protein that enables the detection of low-grade systemic inflammation. As an acute-phase reactant, HS-CRP levels rise in response to tissue injury, infection, or other inflammatory stimuli. In patients with severe stroke, extensive cerebral tissue damage and the associated stress response often trigger a pronounced systemic inflammatory reaction (29). Elevated HS-CRP levels have been consistently associated with increased mortality among stroke patients (30). This elevation reflects a pro-inflammatory state that contributes to endothelial dysfunction, accelerates the progression of atherosclerosis, and activates thrombogenic pathways, thereby exacerbating cerebral ischemic injury and ultimately increasing the risk of stroke-related death (31).

Albumin, a vital nutritional reserve, plays a central role in various physiological processes, including the maintenance of colloidal osmotic pressure, antioxidant activity, anti-inflammatory effects, anticoagulation, and free radical scavenging (32). It serves as both a marker of nutritional status and an indicator of disease severity and has been shown to be closely associated with functional recovery and mortality in stroke patients (33–35). In patients with severe stroke, hypoalbuminemia is common due to a combination of systemic inflammation and disease-related complications. Systemic inflammation can increase metabolic demand and accelerate the catabolism of albumin (36), while proinflammatory cytokines may suppress hepatic albumin synthesis. Moreover, critically ill stroke patients often experience impaired consciousness, dysphagia, gastroparesis, and intestinal barrier dysfunction, all of which contribute to nutrient malabsorption and reduced albumin levels (37). Low serum albumin levels reflect a compromised nutritional and inflammatory status, which may impair tissue repair, weaken immune defense, and increase susceptibility to infections and complications. In addition, reduced oncotic pressure may lead to fluid extravasation and cerebral edema, thereby exacerbating secondary brain injury. The loss of albumin's antioxidant and anti-inflammatory properties further amplifies neuroinflammation, ultimately contributing to worse neurological outcomes and an elevated risk of death.

Neutrophils are among the earliest responders to stroke, with circulating levels increasing within hours of stroke onset. Their accumulation contributes to the disruption of the blood–brain barrier, the development of cerebral edema, and exacerbation of neuronal injury (38). Importantly, elevated neutrophil counts have been shown to correlate with stroke severity, infarct volume, and poorer functional outcomes (39). Although D-Dimer is traditionally regarded as a biomarker of intravascular fibrinolysis and thrombogenesis, elevated levels do not necessarily indicate an increased thrombotic risk. D-dimer is also recognized as an inflammatory marker; during systemic inflammation, fibrin formation followed by secondary fibrinolysis results in the production of D-dimer, and its concentration has been shown to correlate with the severity of the inflammatory response (40). A recent systematic review evaluating the prognostic value of D-dimer in stroke suggested that higher levels are associated with poorer functional outcomes and increased mortality (41). Therefore, monitoring neutrophil and D-dimer levels is of great importance in stroke patients.

Given that critical ill stroke patients often exhibit a high burden of both malnutrition and systemic inflammation, which are strongly associated with poor outcomes, these four biomarkers reflect both nutritional status and inflammatory activity and possess significant prognostic value. Using them in combination may offer a more accurate and comprehensive assessment of this high-risk condition, thereby improving the prediction of adverse outcomes.

The findings of this study show the potential utility of the CAND score as a practical and effective tool for early prognostic assessment in critically ill stroke patients. Since it comprises four routinely available laboratory markers HS-CRP, albumin, neutrophils, and D-Dimer that are regularly measured in standard clinical practice, this composite score can be readily calculated without incurring additional costs or requiring specialized testing. Compared to stroke-specific scores such as A2DS2, CAND is simpler and focuses on systemic inflammation and nutritional status, two critical components influencing prognosis in severe stroke. Furthermore, we have developed an online calculator to facilitate rapid and user-friendly computation of the CAND score, thereby enabling healthcare workers to perform timely risk stratification at the bedside. This may assist healthcare providers in making informed decisions regarding ICU admission, escalation of care, and initiation of more aggressive nutritional or anti-inflammatory interventions. The simplicity and accessibility of the CAND also make it especially valuable in resource-limited settings. Its integration into clinical workflows has the potential to improve prognostic accuracy and support personalized care in stroke management.

### Limitations

This study has several limitations. First, it was a single-center retrospective analysis, and the sample size of the external validation cohort was limited, with only 48 mortality events. Although a *post hoc* power analysis indicated an acceptable statistical power (0.85) to detect the observed effect size, the relatively small number of events may still limit the robustness of the external validation. Second, although 21 nutrition and inflammation related biomarkers were assessed, others—such as prealbumin, IL-6 and erythrocyte sedimentation rate—were not included. Future studies should incorporate a broader biomarker spectrum for more comprehensive profiling. Third, the lack of longitudinal biomarker monitoring precluded assessment of dynamic changes over time. Future research should evaluate biomarker trajectories to enhance prognostic value and guide individualized therapy. Finally, although CAND was designed as a biomarker-based score, we acknowledge that excluding clinical and demographic variables may limit its comprehensiveness. Future studies integrating CAND with clinical predictors may provide a more holistic assessment of patient risk.

## Conclusion

In this study, we developed and validated a novel composite nutrition-inflammation prognostic score (CAND) based on four routinely available biomarkers: HS-CRP, albumin, neutrophils, and D-Dimer. The CAND score demonstrated superior prognostic accuracy and clinical utility for predicting 30-day mortality in critically ill stroke patients. CAND may serve as a practical tool to aid early risk stratification and inform targeted nutritional or therapeutic interventions in critically ill stroke patients.

## Data Availability

The raw data supporting the conclusions of this article will be made available by the authors, without undue reservation.

## References

[1] Feigin VL Owolabi MO World Stroke Organization–Lancet Neurology Commission Stroke Collaboration Group. Pragmatic solutions to reduce the global burden of stroke: a World Stroke Organization-Lancet Neurology Commission. Lancet Neurol. (2023) 22:1160–206. 10.1016/S1474-4422(23)00277-6 37827183 PMC10715732

[2] Cai W Xu J Wu X Chen Z Zeng L Song X. Association between triglyceride-glucose index and all-cause mortality in critically ill patients with ischemic stroke: analysis of the MIMIC-IV database. Cardiovasc Diabetol. (2023) 22:138. 10.1186/s12933-023-01864-x 37312120 PMC10262584

[3] Yang J Peng J Liu G Li F. Predictive value of the random forest model based on bioelectrical impedance analysis parameter trajectories for short-term prognosis in stroke patients. Eur J Med Res. (2024) 29:382. 10.1186/s40001-024-01964-8 39044281 PMC11267791

[4] Kelly PJ Lemmens R Tsivgoulis G. Inflammation and stroke risk: a new target for prevention. Stroke. (2021) 52:2697–706. 10.1161/STROKEAHA.121.034388 34162215

[5] Huang YW Yin XS Li ZP. Association of the systemic immune-inflammation index (SII) and clinical outcomes in patients with stroke: a systematic review and meta-analysis. Front Immunol. (2022) 13:1090305. 10.3389/fimmu.2022.1090305 36591305 PMC9797819

[6] Chen X Hong C Guo Z Huang H Ye L. Association between advanced lung cancer inflammation index and all-cause and cardiovascular mortality among stroke patients: NHANES, 1999-2018. Front Public Health. (2024) 12:1370322. 10.3389/fpubh.2024.1370322 38699426 PMC11063327

[7] Chen Y Yang X Zhu Y Zhang X Ni J Li Y. Malnutrition defined by geriatric nutritional risk index predicts outcomes in severe stroke patients: a propensity score-matched analysis. Nutrients. (2022) 14:4786. 10.3390/nu14224786 36432473 PMC9696179

[8] Liu P Tian H Ji T Zhong T Gao L Chen L. Predictive value of malnutrition, identified via different nutritional screening or assessment tools, for functional outcomes in patients with stroke: a systematic review and meta-analysis. Nutrients. (2023) 15:3280. 10.3390/nu15143280 37513698 PMC10383200

[9] Yuan K Zhu S Wang H Chen J Zhang X Xu P. Association between malnutrition and long-term mortality in older adults with ischemic stroke. Clin Nutr. (2021) 40:2535–42. 10.1016/j.clnu.2021.04.018 33932800

[10] Zhang Z Pereira SL Luo M Matheson EM. Evaluation of blood biomarkers associated with risk of malnutrition in older adults: a systematic review and meta-analysis. Nutrients. (2017) 9:829. 10.3390/nu9080829 28771192 PMC5579622

[11] Germolec DR Shipkowski KA Frawley RP Evans E. Markers of inflammation. Methods Mol Biol. (2018) 1803:57–79. 10.1007/978-1-4939-8549-4_5 29882133

[12] Han X Cai J Li Y Rong X Li Y He L. Baseline objective malnutritional indices as immune-nutritional predictors of long-term recurrence in patients with acute ischemic stroke. Nutrients. (2022) 14:1337. 10.3390/nu14071337 35405949 PMC9000876

[13] Zhu BL Wu YZ Cai ZM Liao CW Sun LQ Liu ZP. A prospective epidemiological analysis of controlling nutritional status score with the poor functional outcomes in Chinese patients with haemorrhagic stroke. Br J Nutr. (2022) 128:192–9. 10.1017/S0007114521003184 34409929

[14] Jiang TT Zhu XY Yin YW Liu HJ Zhang GY. The prognostic significance of malnutrition in older adult patients with acute ischemic stroke. Front Nutr. (2025) 12:1529754. 10.3389/fnut.2025.1529754 39957766 PMC11825317

[15] Yang JX Han YJ Yang MM Gao CH Cao J. Risk factors and predictors of acute gastrointestinal injury in stroke patients. Clin Neurol Neurosurg. (2023) 225:107566. 10.1016/j.clineuro.2022.107566 36603338

[16] Jung SH Hao J Shivakumar M Nam Y Kim J Kim MJ. Development and validation of a novel strong prognostic index for colon cancer through a robust combination of laboratory features for systemic inflammation: a prognostic immune nutritional index. Br J Cancer. (2022) 126:1539–47. 10.1038/s41416-022-01767-w 35249104 PMC9130221

[17] Xiong R Huang H Wu Y Wang S Wang D Ji Z. Incidence and outcome of refeeding syndrome in neurocritically ill patients. Clin Nutr. (2021) 40:1071–6. 10.1016/j.clnu.2020.06.038 32711951

[18] Kwah LK Diong J. National Institutes of Health Stroke Scale (NIHSS). J Physiother. (2014) 60:61. 10.1016/j.jphys.2013.12.012 24856948

[19] Green SM Haukoos JS Schriger DL. How to measure the Glasgow coma scale. Ann Emerg Med. (2017) 70:158–60. 10.1016/j.annemergmed.2016.12.016 28169051

[20] Knaus WA Draper EA Wagner DP Zimmerman JE. APACHE II: a severity of disease classification system. Crit Care Med. (1985) 13:818–29. 10.1097/00003246-198510000-00009 3928249

[21] de Ulíbarri Pérez JI González-Madroño Giménez A González Pérez P Fernández G Rodríguez Salvanés F Mancha Alvarez-Estrada A. [New procedure for the early detection and control of hospital malnutrition]. Nutr Hosp. (2002) 17:179–88. 12395607

[22] Onodera T Goseki N Kosaki G. [Prognostic nutritional index in gastrointestinal surgery of malnourished cancer patients]. Nihon Geka Gakkai Zasshi. (1984) 85:1001–5. 6438478

[23] Chen XL Xue L Wang W Chen HN Zhang WH Liu K. Prognostic significance of the combination of preoperative hemoglobin, albumin, lymphocyte and platelet in patients with gastric carcinoma: a retrospective cohort study. Oncotarget. (2015) 6:41370–82. 10.18632/oncotarget.5629 26497995 PMC4747412

[24] Galizia G Lieto E Auricchio A Cardella F Mabilia A Podzemny V. Naples prognostic score, based on nutritional and inflammatory status, is an independent predictor of long-term outcome in patients undergoing surgery for colorectal cancer. Dis Colon Rectum. (2017) 60:1273–84. 10.1097/DCR.0000000000000961 29112563

[25] Chen P Fang M Wan Q Zhang X Song T Wu S. High-sensitivity modified Glasgow prognostic score (HS-mGPS) is superior to the mGPS in esophageal cancer patients treated with chemoradiotherapy. Oncotarget. (2017) 8:99861–70. 10.18632/oncotarget.21734 29245945 PMC5725136

[26] Yang J Li W Lin X Liang W. A lactate metabolism-related gene signature to diagnose osteoarthritis based on machine learning combined with experimental validation. Aging. (2024) 16:13076–103. 10.18632/aging.205873 39418100 PMC11552637

[27] Ding X Qin J Huang F Feng F Luo L. The combination of machine learning and untargeted metabolomics identifies the lipid metabolism -related gene CH25H as a potential biomarker in asthma. Inflamm Res. (2023) 72:1099–119. 10.1007/s00011-023-01732-0 37081162

[28] Senaviratna NAMR Cooray TMJA. Diagnosing multicollinearity of logistic regression model. Asian J Probab Stat. (2019) 5:1–9. 10.9734/ajpas/2019/v5i230132

[29] Rizo-Téllez SA Sekheri M Filep JG. C-reactive protein: a target for therapy to reduce inflammation. Front Immunol. (2023) 14:1237729. 10.3389/fimmu.2023.1237729 37564640 PMC10410079

[30] Liu F Yang P Wang Y Shi M Wang R Xu Q. HS-CRP modifies the prognostic value of platelet count for clinical outcomes after ischemic stroke. J Am Heart Assoc. (2023) 12:e30007. 10.1161/JAHA.123.030007 37449575 PMC10382093

[31] Chen L Wang M Yang C Wang Y Hou B. The role of high-sensitivity C-reactive protein serum levels in the prognosis for patients with stroke: a meta-analysis. Front Neurol. (2023) 14:1199814. 10.3389/fneur.2023.1199814 37342777 PMC10278886

[32] Soeters PB Wolfe RR Shenkin A. Hypoalbuminemia: pathogenesis and clinical significance. JPEN J Parenter Enteral Nutr. (2019) 43:181–93. 10.1002/jpen.1451 30288759 PMC7379941

[33] Singer P Blaser AR Berger MM Alhazzani W Calder PC Casaer MP. ESPEN guideline on clinical nutrition in the intensive care unit. Clin Nutr. (2019) 38:48–79. 10.1016/j.clnu.2018.08.037 30348463

[34] Arques S. Serum albumin and cardiovascular disease: state-of-the-art review. Ann Cardiol Angeiol. (2020) 69:192–200. 10.1016/j.ancard.2020.07.012 32797938

[35] Thuemmler RJ Pana TA Carter B Mahmood R Bettencourt-Silva JH Metcalf AK. Serum albumin and post-stroke outcomes: analysis of UK regional registry data, systematic review, and meta-analysis. Nutrients. (2024) 16:1486. 10.3390/nu16101486 38794724 PMC11124370

[36] Wang H Ye J. Regulation of energy balance by inflammation: common theme in physiology and pathology. Rev Endocr Metab Disord. (2015) 16:47–54. 10.1007/s11154-014-9306-8 25526866 PMC4346537

[37] Yuan F Yang F Zhang W Jia Y Ma Y Qu Y. Optimizing early enteral nutrition in severe stroke (OPENS): protocol for a multicentre randomized controlled trial. BMC Neurol. (2019) 19:24. 10.1186/s12883-019-1253-2 30755171 PMC6371599

[38] Denorme F Rustad JL Campbell RA. Brothers in arms: platelets and neutrophils in ischemic stroke. Curr Opin Hematol. (2021) 28:301–7. 10.1097/MOH.0000000000000665 34183536 PMC8483595

[39] Tang C Wang C Zhang Y Xue L Li Y Ju C. Recognition, intervention, and monitoring of neutrophils in acute ischemic stroke. Nano Lett. (2019) 19:4470–7. 10.1021/acs.nanolett.9b01282 31244234

[40] Franchini M Focosi D Pezzo MP Mannucci PM. How we manage a high D-dimer. Haematologica. (2024) 109:1035–45. 10.3324/haematol.2024.285769 37881856 PMC10985443

[41] Ohara T Farhoudi M Bang OY Koga M Demchuk AM. The emerging value of serum D-dimer measurement in the work-up and management of ischemic stroke. Int J Stroke. (2020) 15:122–31. 10.1177/1747493019876538 31537182

