# Supplementary material for: Development and validation of a novel nutrition-inflammation prognostic score for predicting 30-day mortality in critically ill stroke patients
Source: Front Nutr. 2025 Sep 3;12:1658896. doi: 10.3389/fnut.2025.1658896 (PMC12442555; doi:10.3389/fnut.2025.1658896)
Supplement: Supplementary file 1 [file Data_Sheet_1.pdf]

Supplementary Table 1. Details of six Nutrition-Inflammation prognostic scores.

Nutrition risk screening tools	Evaluation content	Cut-off value
CONUT	Albumin, lymphocyte count, and total cholesterol levels	≥2
PNI	Formula=10×albumin(g/dl) +0.005 total lymphocyte count (per mm3)	<45
HALP	Formula= [hemoglobin (g/L) × albumin (g/L) × lymphocytes (/L)]/platelets (/L)]	< 56.8
NPS	Albumin, total cholesterol, neutrophil-lymphocyte ratio, Lymphocyte-Monocyte Ratio	≥1
PINI	Formula=[albumin (g/dL) × 0.9] - [monocyte (mm3) × 0.0007]	<3
HS-m GPS	Albumin, high-sensitivity C-reactive protein	≥1

Abbreviations: CONUT, controlling nutritional status score; PNI, prognostic nutritional index; HALP, Albumin, Lymphocyte, Platelet Score; NPS, Naples Prognostic Score; HS-m GPS,high-sensitivity modified Glasgow Prognostic Score; PINI, prognostic immune nutritional index.

Supplementary Table 2. Multivariable Logistic Regression Identifying Predictors Included in the CAND Score

Variable	Coef	P	OR	95%CI
HS-CRP	0.0358	0.009	1.036	1.009-1.065
Albumin	-0.0507	0.005	0.951	0.918-0.984
Neutrophils	0.0861	0.001	1.090	1.035-1.147
D-Dimer	0.2388	<0.001	1.270	1.189-1.356

Supplementary Table 3. Unadjusted Association Between High Nutrition-Inflammation Risk and 30-day Mortality (Model 1).

High risk group	Training cohort (n = 725)		Validation cohort (n =201)	
	Hazard Ratio (95% CI)	p-value	Hazard Ratio (95% CI)	p-value
CAND	5.894(4.414-7.87)	<0.001	4.633(2.613-8.214)	<0.001
PNI	1.595(1.201-2.119)	0.001	1.994(1.131-3.514)	0.017
CONUT	1.632(1.127-2.363)	0.01	1.743(0.888-3.42)	0.106
HALP	1.582(0.985-2.54)	0.058	0.959(0.449-2.049)	0.914
NPS	2.15(1.137-4.063)	0.018	1.389(0.691-2.792)	0.356
PINI	1.488(1.063-2.082)	0.021	2.589(1.366-4.906)	0.004
HS-m GPS	3.121(2.09-4.659)	<0.001	2.053(1.045-4.032)	0.037

Abbreviations: CONUT, controlling nutritional status score; PNI, prognostic nutritional index; HALP, Albumin, Lymphocyte, Platelet Score; NPS, Naples Prognostic Score; HS-m GPS,high-sensitivity modified Glasgow Prognostic Score; PINI, prognostic immune nutritional index.

Supplementary Table 4. Multivariate Cox Regression Analysis Adjusted for Age and Sex of the Association Between High Nutrition-Inflammation Risk and 30-Day Mortality (Model 2).

High risk group	Training cohort (n = 725)		Validation cohort (n = 201)	
	Hazard Ratio (95% CI)	p-value	Hazard Ratio (95% CI)	p-value
CAND	5.86(4.386-7.831)	<0.001	4.667(2.623-8.304)	<0.001
PNI	1.42(1.066-1.892)	0.017	1.776(0.996-3.166)	0.052
CONUT	1.429(0.981-2.08)	0.063	1.783(0.908-3.5)	0.093
HALP	1.494(0.93-2.401)	0.097	0.847(0.391-1.832)	0.673
NPS	2.128(1.126-4.024)	0.02	1.443(0.717-2.904)	0.304
PINI	1.322(0.942-1.856)	0.106	2.52(1.324-4.798)	0.005
HS-m GPS	2.98(1.994-4.453)	<0.001	2.162(1.099-4.251)	0.025

Abbreviations: CONUT, controlling nutritional status score; PNI, prognostic nutritional index; HALP, Albumin, Lymphocyte, Platelet Score; NPS, Naples Prognostic Score; HS-m GPS,high-sensitivity modified Glasgow Prognostic Score; PINI, prognostic immune nutritional index.

Supplementary Table 5. Association Between High Nutrition-Inflammation Risk and 30-Day Mortality: A Multivariate Cox Regression Analysis (Model 3)

High risk group	Training cohort (n = 725)		Validation cohort (n = 201)	
	Hazard Ratio (95% CI)	p-value	Hazard Ratio (95% CI)	p-value
CAND	3.273(2.413-4.437)	<0.001	3.608(1.888-6.894)	<0.001
PNI	1.283(0.947-1.74)	0.108	1.544(0.836-2.853)	0.166
CONUT	0.759(0.508-1.133)	0.178	1.577(0.745-3.341)	0.234
HALP	0.963(0.593-1.565)	0.88	0.598(0.236-1.515)	0.279
NPS	1.906(0.977-3.716)	0.058	1.518(0.702-3.283)	0.289
PINI	0.916(0.639-1.312)	0.631	1.915(0.930-3.946)	0.078
HS-m GPS	1.875(1.24-2.836)	0.003	2.201(1.035-4.683)	0.04

Abbreviations: CONUT, controlling nutritional status score; PNI, prognostic nutritional index; HALP, Albumin, Lymphocyte, Platelet Score; NPS, Naples Prognostic Score; HS-m GPS,high-sensitivity modified Glasgow Prognostic Score; PINI, prognostic immune nutritional index.

Supplementary Table 6. Comparison of Net Reclassification Improvement (NRI) and Integrated Discrimination Improvement (IDI) for Mortality Prediction in Training and Testing Cohorts

**Model**	**Training cohort**		**Testing cohort**		**Training cohort**		**Testing cohort**	
	NRI	p-value	NRI	p-value	IDI	p-value	IDI	p-value
Baseline model	Reference							
Baseline+ CAND	0.252	<0.001	0.194	0.033	0.098	<0.001	0.113	<0.001
Baseline+ PNI	0.014	0.493	0.098	0.193	0.024	<0.001	0.028	0.001
Baseline+ CONUT	0.010	0.741	0.052	0.482	0.026	<0.001	0.038	<0.001
Baseline+ HALP	0.025	0.006	0.121	0.139	0.004	<0.001	0.038	<0.001
Baseline+ NPS	0.014	0.772	0.068	0.414	0.080	<0.001	0.039	<0.001
Baseline+ PINI	0.017	0.0368	0.076	0.303	0.003	<0.001	0.040	0.002
Baseline+ HS-m GPS	0.062	0.126	0.017	0.872	0.059	<0.001	0.081	<0.001

Abbreviations: CONUT, Controlling Nutritional Status; PNI, Prognostic Nutritional Index; HALP, Hemoglobin-Albumin-Lymphocyte-Platelet; NPS, Naples Prognostic Score; HS-m GPS, high-sensitivity modified Glasgow Prognostic Score; PINI, Prognostic Immune-Nutritional Index; NRI, net reclassification improvement; IDI, integrated discrimination improvement. The baseline model incorporated age, sex, BMI, living situation, smoking status, alcohol consumption, stroke subtype and comorbidities (hypertension, diabetes mellitus, COPD, coronary artery disease), and admission scores: NIHSS, GCS, and APACHE II.

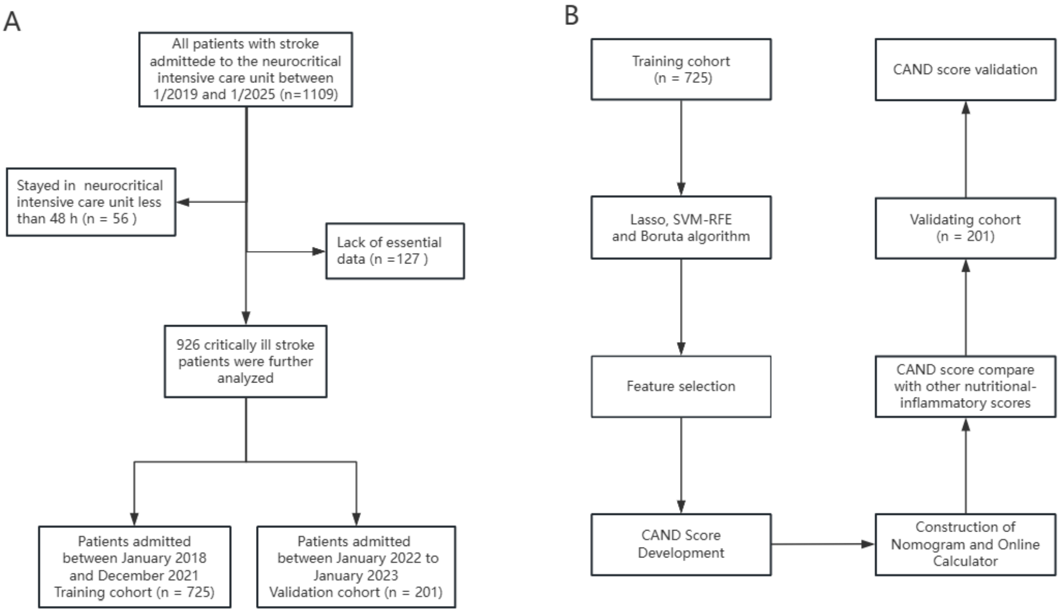

Supplementary Figure1. Flow chart of the patient inclusion process.

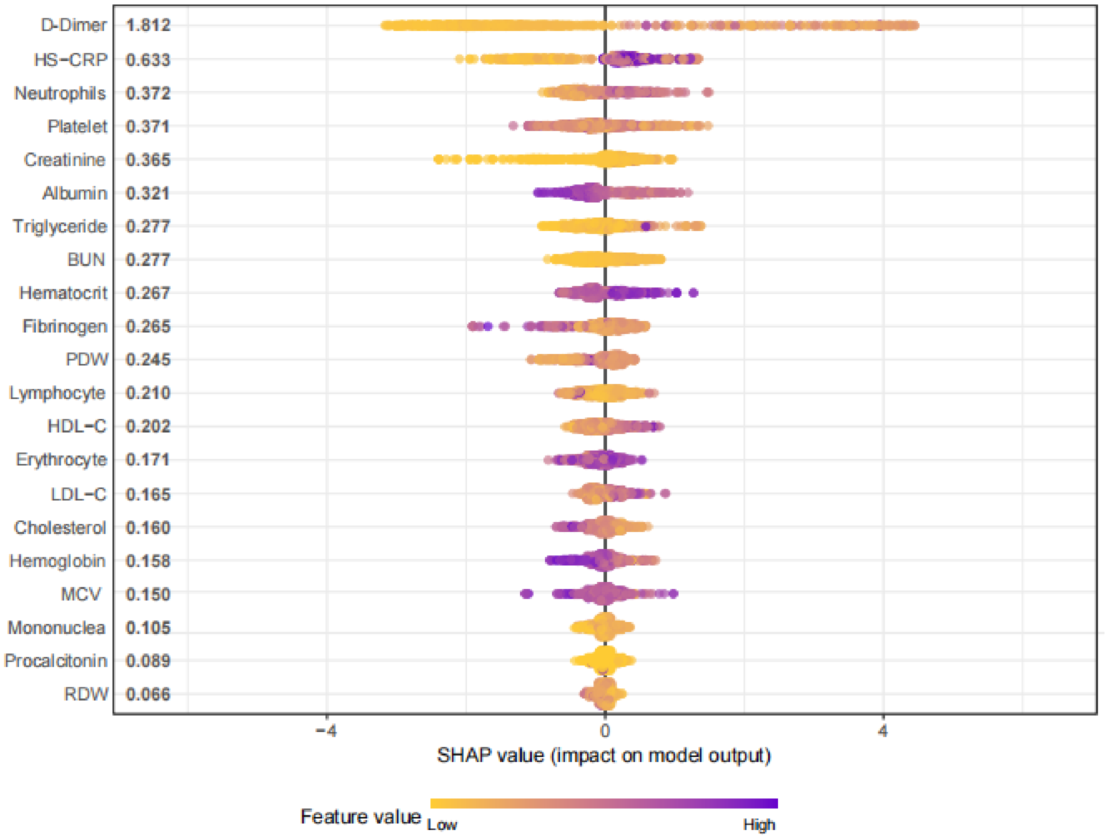

Supplementary Figure 2. SHAP Summary Plot of Nutritional and Inflammatory Biomarkers for Mortality Risk Prediction

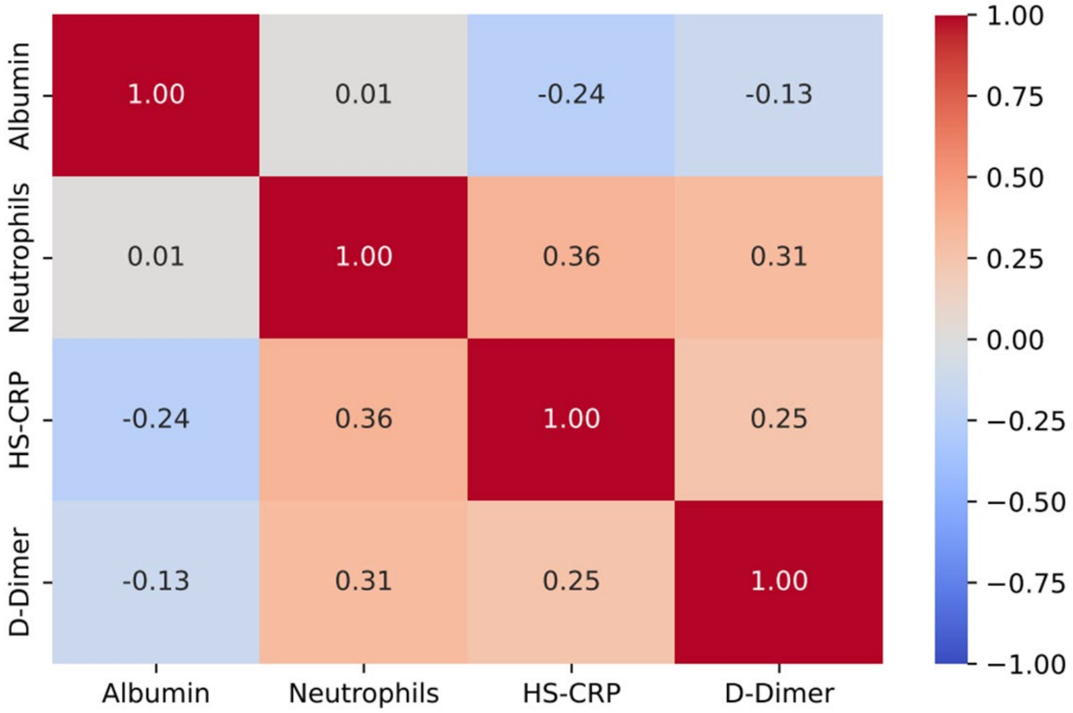

Supplementary Figure 3. Heatmap of Pairwise Pearson Correlation Coefficients Among Selected Features.

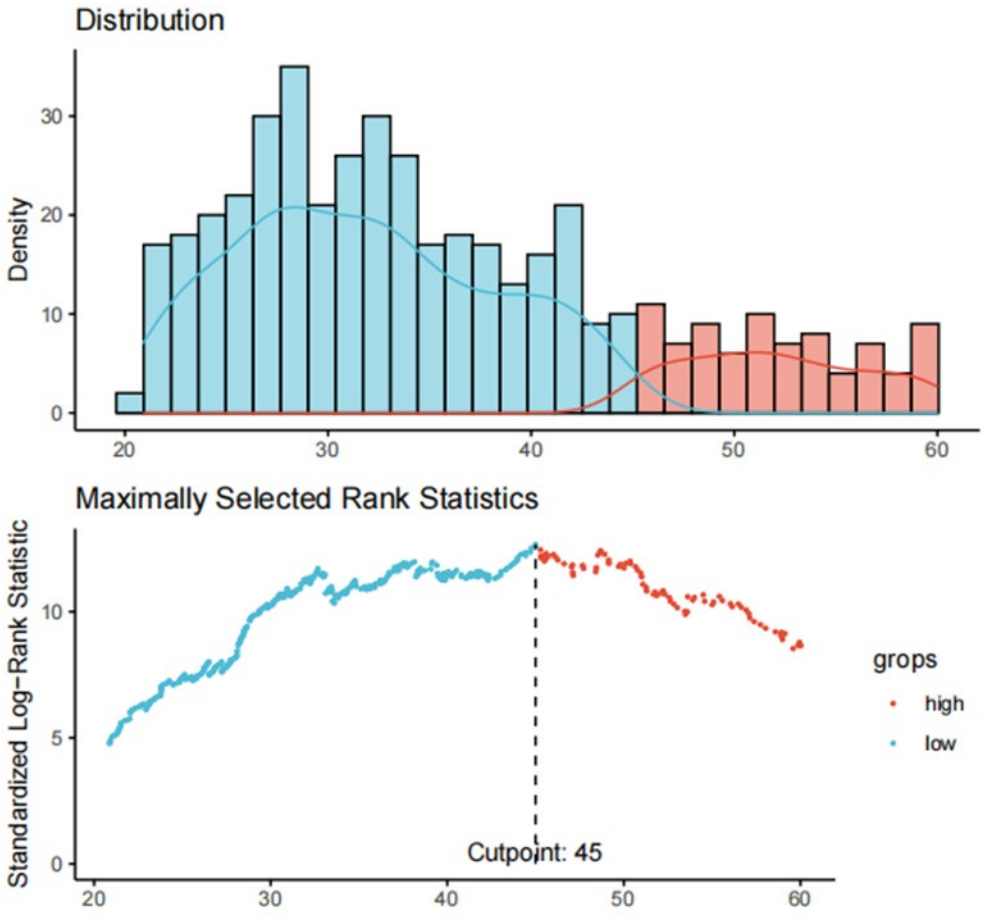

Supplementary Figure 4. Identification of the Optimal CAND Cut-off Using Log-Rank Test.

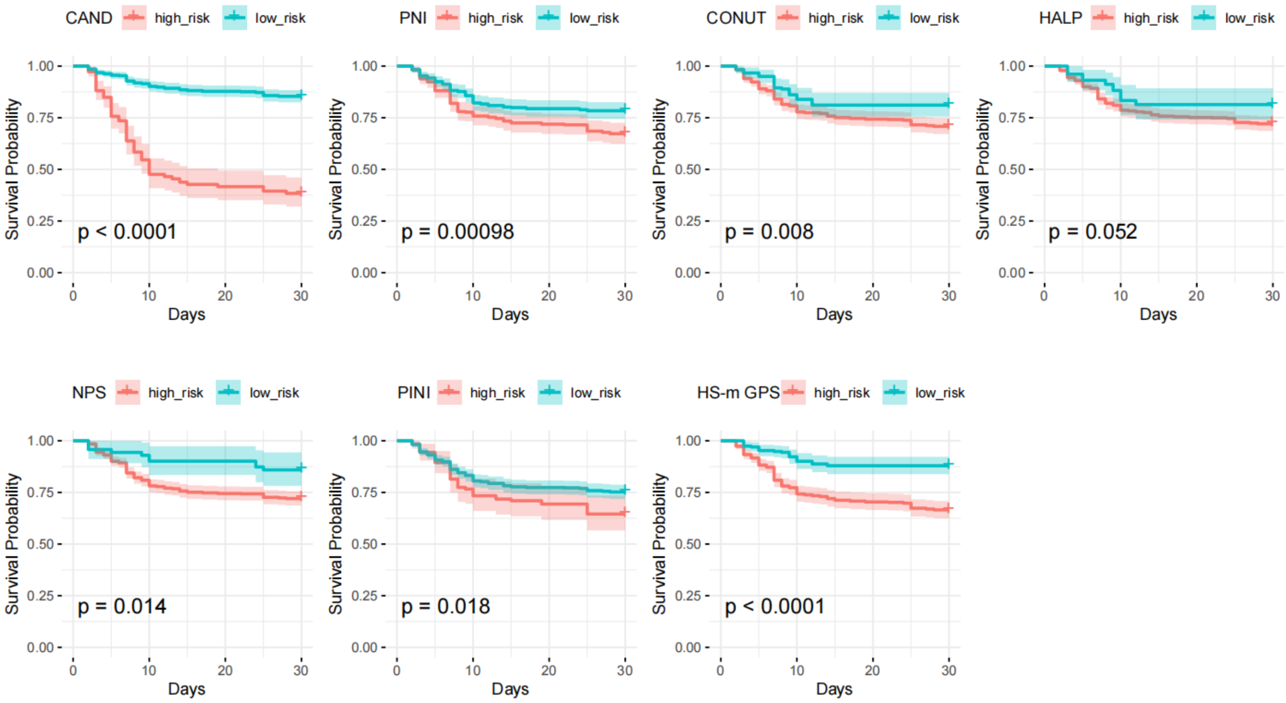

Supplementary Figures 5. Kaplan－Meier Survival Curves According to Nutrition-Inflammation Risk in the Training Cohort

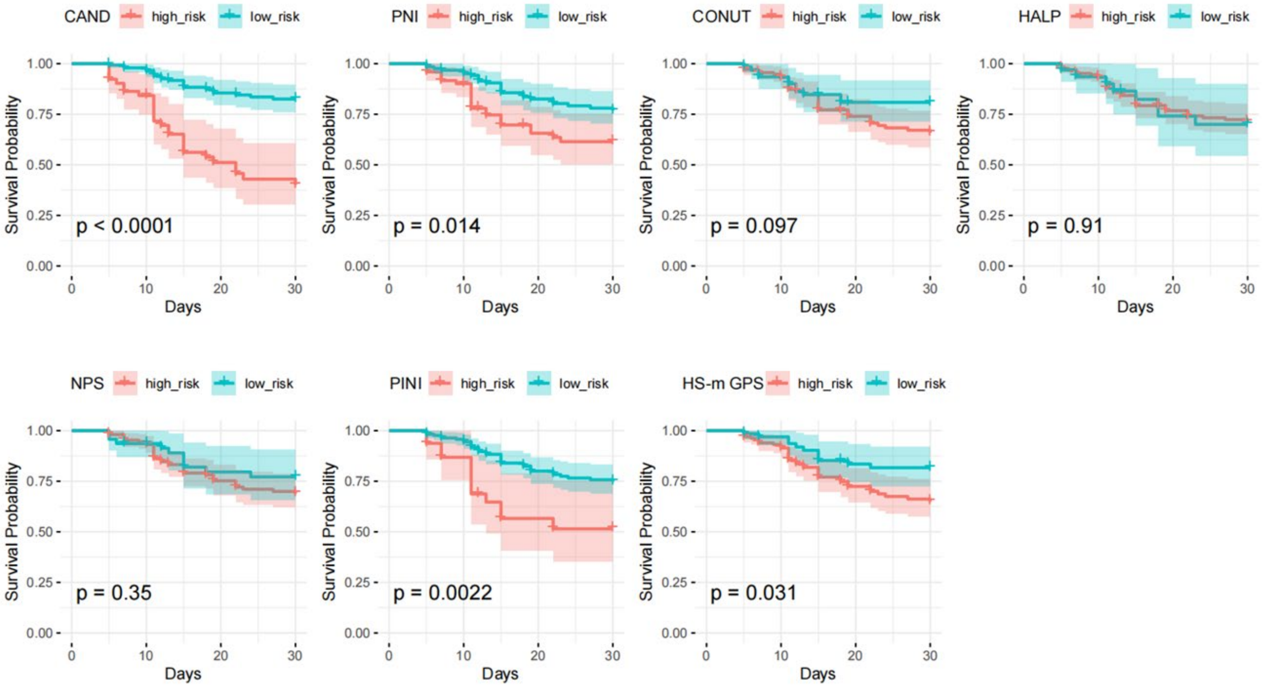

Supplementary Figures 6. Kaplan－Meier Survival Curves According to Nutrition-Inflammation Risk in the Validation Cohort
